# Impaired Clearance of Early Apoptotic Cells Mediated by Inhibitory IgG Antibodies in Patients with Primary Sjögren's Syndrome

**DOI:** 10.1371/journal.pone.0112100

**Published:** 2014-11-14

**Authors:** Menelaos N. Manoussakis, George E. Fragoulis, Aigli G. Vakrakou, Haralampos M. Moutsopoulos

**Affiliations:** 1 Department of Pathophysiology, School of Medicine, University of Athens, Athens, Greece; 2 Hellenic Pasteur Institute, Athens, Greece; Keio University School of Medicine, Japan

## Abstract

**Objectives:**

Deficient efferocytosis (i.e. phagocytic clearance of apoptotic cells) has been frequently reported in systemic lupus erythematosus (SLE). Todate, patients with primary Sjögren's syndrome (SS) have not been assessed for phagocytosis of apoptotic cells (ApoCell-phagocytosis) and of particulate targets (microbeads, MB-phagocytosis).

**Design:**

ApoCell-phagocytosis and MB-phagocytosis were comparatively assessed by flow cytometry in peripheral blood specimens and monocyte-derived macrophage (MDM) preparations from healthy blood donors (HBD) and consecutive SS, SLE and rheumatoid arthritis (RA) patients. Cross-admixture ApoCell-phagocytosis experiments were also performed using phagocytes from HBD or patients, and apoptotic cells pretreated with whole sera or purified serum IgG derived from patients or HBD.

**Results:**

Compared to HBD, approximately half of SS and SLE patients studied (but not RA) manifested significantly reduced ApoCell-phagocytosis (p<0.001) and MB-phagocytosis (p<0.003) by blood-borne phagocytes that correlated inversely with disease activity (p≤0.004). In cross-admixture assays, healthy monocytes showed significantly reduced ApoCell-phagocytosis when fed with apoptotic cells that were pretreated with sera or purified serum IgG preparations from SS and SLE patients (p<0.0001, compared to those from HBD or RA). Such aberrant effect of the SS and SLE sera and IgG preparations correlated linearly with their content of IgG antibodies against apoptotic cells (p≤0.0001). Phagocytic dysfunction maybe also present in certain SS and SLE patients, as supported by deficient capacity of MDM for ApoCell-phagocytosis and MB-phagocytosis under patients' serum-free conditions.

**Conclusion:**

Similarly to SLE, efferocytosis is frequently impaired in SS and is primarily due to the presence of inhibitory IgG anti-ApoCell antibodies and secondarily to phagocytes' dysfunction.

## Introduction

Apoptosis represents a major mechanism of programmed cell death that is essential for the regulation of tissue growth and homeostasis [1]. Normally, cells dying by apoptosis undergo specific changes that target them for rapid clearance by professional phagocytes, such as macrophages. This process leads to the active production of anti-inflammatory mediators by phagocytes and thus facilitates the “immunologically silent” removal of apoptotic cells [2]. The prompt elimination of apoptotic cells (also termed “efferocytosis”) [3] is a very crucial biological process, since lingering apoptotic cells eventually proceed to the state of “late apoptosis” or “secondary necrosis” wherein they may contribute to inflammatory reactions via the release of immunogenic intracellular components, including modified autoantigens and “danger signals” [4]. In fact, apoptosis and efferocytosis act in concert to regulate various processes, such as embryogenesis, tissue homeostasis, tolerance the elimination of damaged cells, and the resolution of inflammation [5]–[7].

The occurrence of defective efferocytosis in certain inflammatory diseases is thought to have pathogenetic significance, based on the pro-inflammatory potential of secondary necrotic cells [8] Among them, systemic lupus erythematosus (SLE) is regarded as the archetypical disease model where the impaired clearance of apoptotic cells by macrophages represents a possible mechanism for the development of chronic autoimmune reactions and organ damage [9]–[11]. Apart from defective efferocytosis, various in vitro clearance defects of macrophages have been described in SLE, including aberrant Fc-gamma receptor-mediated uptake of IgG ligand-coated erythrocytes [12] and decreased phagocytosis of yeast cells [13] and particulate targets [10]. These aberrations have been attributed to intrinsic defects of patients' phagocytes [9], to the decreased density of circulating macrophages [14], as well as to the effect of serum components [11], [15]–[17].

Primary Sjögren's syndrome (SS), which is characterized by mononuclear cell infiltrates in exocrine glands and parenchymal organs, shares several immunologic manifestations with SLE. These include various features of B-cell hyperactivity, such as the profound hypergammaglobulinemia, multiple autoantibodies, circulating immune complexes and evidence of complement consumption [18]. In this context, we presently sought to comparatively investigate the capacity of peripheral blood monocytes and monocyte-derived macrophages (MDM) of SS and SLE patients for phagocytosis of apoptotic cells and of particulate targets. For this purpose, we established ex-vivo phagocytosis assays and assessed patients with SLE, SS and RA, as well as healthy individuals. Our findings indicate that considerable proportions of SS and SLE patients (but not RA) manifest deficient phagocytosis of apoptotic cells and of particulate targets that correlate with the activity of these diseases, and apparently owes primarily to inhibitory IgG anti-ApoCell antibodies and secondarily to the dysfunction of phagocytes.

## Patients and Methods

### Patients

Specimens of peripheral blood were obtained after informed consent from 43 consecutive unselected Greek Caucasian patients with primary-SS, 27 with SLE and 14 with RA [19]–[21] (**Table 1** **)**, as well as from Greek Caucasian healthy blood donors (2 groups; HBD-1 and HBD-2, 17 each, age- and sex-matched to the SS and SLE groups, respectively). In all assays, HBD-1 and HBD-2 groups exhibited similar results, without statistical difference to each other. Thus, they were subsequently considered as a single group (HBD) in the comparative analyses. The study was approved by the Medical council of “Laikon” University Hospital (Research Ethics Committee). Written informed consent was given by all individuals for their participation in the study, as well as for the usage of their clinical records. Patients' medical records were retrospectively analyzed for demographic variables, clinical and laboratory features. At the time of the study, patients studied were assessed for disease activity by calculation of ESSDAI (for SS) [22], SLEDAI (for SLE), DAS28 (for RA) and for disease severity by SSDDI (for SS) [23], SLICC-ACR (for SLE) [24] and by global disease severity index (for RA). None of the SLE or RA patients studied manifested any evidence of secondary or associated Sjögren's syndrome. SS patients were also analyzed for the presence of extraglandular manifestations and of type-I disease, as previously [25]. At the time of investigation, none of the patients or controls studied displayed evidence of infection or had history of infection with hepatitis viruses or HIV. Sera were tested for anti-Ro/SSA, anti-La/SSB by counter-immunoelectrophoresis, for anti-C1q, anti-native histone and anti-chromatin antibodies by ELISA (QuantaLite), for C3 and C4 complement levels by radial immunodiffusion and for C1q levels by nephelometry. Total immunoglobulin IgG was isolated from serum samples of patients and HBD using the Melon Gel purification Kit (Pierce), according to the manufacturer's protocol, aliquoted and stored at −20°C. Protein yields were quantified spectrophotometrically and purification was routinely found to be more than 95% by SDS-PAGE electrophoresis.

**Table 1:** Selected anthropometric, clinical and laboratory features of the patients studied.

Disease Features	SS (n = 43)	SLE (n = 27)	RA (n = 14)
Age, years, median (range)	52.5 (33–67)	37.5 (19–45)	60.0 (38–75)
Sex, female:male	43∶0	25∶2	7∶7
Disease duration, years, median (range)	11 (2–23)	11 (1–20)	10 (1–17)
Sicca man manifestations, no. positive (%)	43 (100)	0 (0.0)	0 (0.0)
Disease activity, median (range) a	9 (0–41)	4 (0–16)	5.2 (3.5–7.0)
Disease severity, median (range) a	2 (2–11)	2 (0–6)	2.9 (1.3–5.4)
Type-I SS disease, no. positive (%) a	25 (58.1)	NA	NA
ANA, titer^-1^, median (range)	320 (0–2560)	640 (0–2560)	0
Anti-Ro/SSA, no. positive (%)	29 (67.4)	15 (40.5)	0 (0.0)
Anti-La/SSB, no. positive (%)	13 (30.2)	0 (0.0)	0 (0.0)
Anti-dsDNA, no. positive (%)	0 (0.0)	18 (47.4)	0 (0.0)
Anti-chromatin, no. positive (%)	0/27 (0.0)	4/19 (21.1)	0 (0.0)
Anti-histone, no. positive (%)	0/27 (0.0)	5/19 (26.3)	0 (0.0)
Low serum C3 and/or C4, no. positive (%) b	18 (41.9)	23 (60.5)	0 (0.0)
Low serum C1q, no. positive (%) c	5/17 (29.4)	5/15 (33.3)	ND
Rheumatoid factor, no. positive (%)	20 (46.5)	ND	11 (78.6)

a: disease activity, disease severity and Type-I SS disease were defined as described in Patients and Methods,

b: low serum C3<90 mg/dl and C4<20 mg/dl,

c: low serum C1q<15 mg/dl, NA: not applicable, ND: not done.

### Generation of early apoptotic cells

The Jurkat cell line (ATCC) was grown in RPMI-1640 medium (Gibco) supplemented with 10% heat-inactivated human AB-serum and 0.1% gentamicin. Apoptosis was induced in Jurkat cells by exposure to Ultraviolet-B irradiation (800 mJ/cm^2^) at room temperature using Stratalinker-1800 (Stratagene), as previously [26]. Subsequently, cells were washed twice with PBS, seeded into Petri dishes 60×15 mm in the above culture medium (0.7×10^7^ cells/mL) and incubated for 4-hrs at 37°C. The percentage of apoptotic cells was determined by flow cytometry (Facscalibur, BD) using annexin-V/propidium iodide staining (R&D). As determined in extensive preliminary experiments and verified at each preparation, after the 4-hrs incubation, approximately 60% of Jurkat cells were early apoptotic (annexin-V-positive/propidium iodide-negative, mean ±SEM: 61.2±5.0%, n = 10 experiments), with the remaining of cells being viable (negative for both annexin-V and propidium iodide, 37.5±4.3%). In such preparations, late apoptotic cells (positive for both annexin-V and propidium iodide) were typically <2% of cells (1.9±0.2%).

### Assessment of serum IgM and IgG immunoglobulin binding to apoptotic cells

The levels of IgM and IgG antibodies to early apoptotic cells (anti-ApoCell) were assayed in serum specimens (diluted to 20% and 5% v/v, respectively) by flow cytometry as previously [16], using early apoptotic Jurkat cells prepared as above and FITC-labeled rabbit antisera to human IgM or IgG immunoglobulin as secondary antibody (Dako), respectively. In each experiment, the reproducibility and inter-assay variation was evaluated by testing two standard specimens (aliquoted and stored at 20°C) in duplicates consisted of pooled sera from 3 healthy donors (negative control) and 3 SLE patients (positive control), respectively. For the estimation of ApoCell-specific serum IgM or IgG antibody binding, the non-specific binding of the secondary antibody was considered as baseline, the values obtained were normalized to the standard negative control specimen and the results were expressed as binding index (percent gated by mean fluorescence intensity). Coefficient variation for standard specimens was less than 12.5% between different experiments. The occurrence of anti-ApoCell antibodies in purified IgG preparations (50 µg/mL) of patients and controls was also assessed by flow cytometry as above.

### Assessment of phagocytosis of apoptotic cells (ApoCell-phagocytosis) by peripheral blood monocytes

For the assessment of ApoCell-phagocytosis by peripheral blood phagocytes, freshly prepared early apoptotic Jurkat cells were labeled with the fluorescent dye carboxylfluorescein diaceteate-succinimidylester (CFSE, Molecular Probes) and were added (1×10^6^ cells) in duplicate samples (100 µl) of freshly drawn heparinized peripheral blood. Mixtures were incubated for 90-min at 37°C and subsequently, erythrocytes were destroyed with a lysis buffer (Pharmingen). In preliminary experiments, using fluorescence microscopy, normal peripheral blood monocytes and polymorphonuclear cells were found to ingest appreciable amounts of CFSE-stained apoptotic cells, but not viable cells. ApoCell-phagocytosis by monocytes was quantitatively assessed in peripheral blood samples from HBD and patients by flow cytometry, using the appropriate forward/side scatter pattern gate and confirmation by staining with CD14 monoclonal antibody (Santa Cruz). Monocytes were electronically gated, and the uptake of fluorescent CFSE-stained apoptotic Jurkat cells by these cells was estimated as ApoCell-phagocytosis index (ApoCell-PhI), which was calculated as the product of the percentage of gated fluorescent-positive cells by their mean fluorescence intensity. The localization of apoptotic Jurkat cells in the gate of granulocytes had precluded the assessment of ApoCell-phagocytosis by the latter type of cells.

### ApoCell-phagocytosis by monocyte-derived macrophages (MDM)

For the preparation of MDM, mononuclear cells were isolated from peripheral blood of patients and healthy donors by density-gradient centrifugation (BD-Vacutainer), washed thrice with HBSS (Gibco) and plated onto 24-well plates (Corning, 4×10^6^ cells/well) in DMEM (Gibco) supplemented with 10% heat-inactivated FBS. Following 1-hour incubation at 37°C, 5% CO_2_, non-adherent cells were removed by washing with HBSS and the adherent monocytes were allowed to mature to macrophages by cultivation for 8-days in X-VIVO-10 medium (Lonza) containing 10% heat-inactivated AB human serum, as previously [26]. Culture medium was changed at day-3, and following an additional 5-day culture, the supernatant was aspirated and fresh preparations of early apoptotic cells were added in each well, in X-VIVO-10 (3×10^6^ cells/ml). MDM were washed thrice with cold PBS to remove the non-ingested apoptotic cells, were detached with EDTA-lidocaine solution and ApoCell-phagocytosis was analyzed by flow cytometry, as above. MDM preparations were typically 50-65% CD14-positive.

### Cross-admixture experiments for the assessment of the influence of serum factors in ApoCell-phagocytosis

To assess the influence of serum components in ApoCell-phagocytosis, apoptotic cells were pre-treated (for 15-min at 37°C) with DMEM culture medium alone, with whole serum or with purified IgG preparations (50 µg/ml) derived from autoimmune patients or HBD. Apoptotic cells were washed and incubated (1×10^6^ cells) with preparations of isolated peripheral blood mononuclear cells (1×10^6^ cells) or MDM (1×10^5^ cells) derived from a healthy donor, and the samples were analyzed for ApoCell-phagocytosis, as above. In separate experiments, prior the addition to mixtures, serum samples from HBD were heat-inactivated (56°C, 30-min) to destroy complement. The rates of ApoCell-phagocytosis by healthy MDM of apoptotic cells pretreated with purified IgG preparations (50 µg/ml) derived from either autoimmune patients or HBD were also comparatively investigated by two-color flow cytometry analyses of healthy MDM stained with CD14-PE (BD-Pharmingen) and CFSE-labelled IgG-pretreated apoptotic cells, as well as by single-color flow cytometry using apoptotic cells labelled with the pH-sensitive fluorescent dye pHrodo succinimidyl ester (pHrodo-SE, Life Technologies, 20 ng/ml at room temperature for 30-min), which allows the actual detection of ingested apoptotic cells owing to increased light emission in the acidic environment of the phagosomes of phagocytes [27].

### Phagocytosis assay of fluorescent microbeads (MB-phagocytosis) by peripheral blood phagocytes and MDM

Fluorescent monodisperse polystyrene microbeads (MB) with 1-µm diameter (Fluoresbrite YG, Polysciences) were used. The experimental conditions were optimized in preliminary experiments, where the ingestion of MB was verified by fluorescent microscopy. In such experiments, the application of uncoated microbeads or microbeads coated with human immunoglobulin or human serum albumin had yielded equivalent results, whereas no discernible uptake was observed upon incubation of blood samples with microbeads at 4°C. In brief, duplicate samples of heparinized peripheral blood (200 µl) were mixed with MB (4×10^7^ beads/ml) and were incubated for 30-min at 37°C. Erythrocytes were lysed as above, and following three washings with cold PBS to remove the non-ingested MB, the samples were analyzed by flow cytometry. Typical laser scatter properties were used to determine separate acquisition gates for granulocytes and monocytes. MB-phagocytosis index (MB-PhI) was calculated as the product of the percentage of gated fluorescence-positive cells by their mean fluorescence intensity. MB-phagocytosis by MDM preparations was also assessed as above.

### Statistical analyses

Correlations were calculated using the Spearman's rank correlation coefficient. Group comparisons were performed by Mann-Whitney rank sum test. For paired comparisons, Wilcoxon signed-rank test and one-way analysis of variance were used, when appropriate. Analyses were conducted using SPSS 15.0 and Graph Pad 5.0 softwares. The results were expressed as median and range, and correlations with two-tailed p-values less than 0.05 were considered statistically significant.

## Results

### Serological aberrations in SS and SLE patients studied

Serological analyses had revealed several aberrations, including the presence of various anti-nuclear autoantibodies in the serum samples from the SS and SLE patients studied, but not in those from RA (**Table 1**).

### Significantly impaired ApoCell-phagocytosis by peripheral blood monocytes of SS and SLE patients

**Figure 1:**
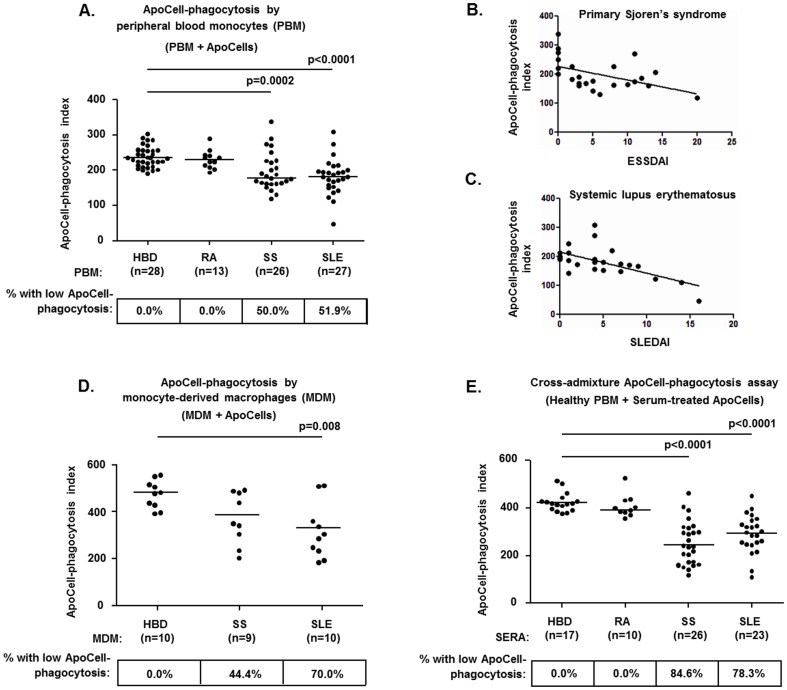
The peripheral blood (PB) monocytes and monocyte-derived macrophages (MDM) of SS and SLE patients manifest significantly impaired ApoCell-phagocytosis. The aberrant uptake of apoptotic cells by blood-borne phagocytes largely resides in the patients' sera. **A.** Significantly decreased ApoCell-phagocytosis by PB monocytes in SS and SLE patients, but not in RA. **B–C.** The ApoCell-phagocytosis index values observed in SS and SLE patients correlated inversely with the disease activity indices of these diseases. **D.** Decreased ApoCell-phagocytosis by MDM in SS and SLE patients. **E.** Cross-admixture experiments illustrating the significantly reduced capacity of sera from SS and SLE patients to support ApoCell-phagocytosis by normal peripheral blood (PB) monocytes, in contrast to sera from HBD and from RA patients. In panels A, C and D the horizontal lines indicate the median levels in each group, whereas the numbers in boxes indicate the percentages of individuals with decreased ApoCell-phagocytosis, as defined by the presence of ApoCell-PhI values that were two standard deviations below the corresponding mean of HBD. Statistically significant comparisons of patient groups to HBD are shown. In panel B, the mean ApoCell-PhI values of SS-derived MDM were marginally different compared to MDM (p = 0.06).

The study of ApoCell-phagocytosis had yielded similar results between RA patients and HBD, but significantly decreased ApoCell-PhI values in both SS and SLE patient groups, compared to HBD (p = 0.0002 and p<0.0001, respectively) and to RA patients (p = 0.0009 and p = 0.0004, respectively) (**Figure 1A**). Conversely, ApoCell-PhI values were not statistically different between SS and SLE patients. Decreased ApoCell-phagocytosis was observed in approximately half of SS and SLE patients, compared to none of RA patients studied (**Figure 1A**).

Among SS patients, ApoCell-phagocytosis by monocytes (ApoCell-PhI values) was found to correlate inversely with ESSDAI scores (r = −0.559, p = 0.004, **Figure 1B**) and to be significantly impaired among patients with type-I disease (in type-I disease; median: 168, range: 119–206, in type-II disease; median: 221, range: 130–338, p = 0.04) or with anti-Ro/SSA autoantibodies (in anti-Ro/SSA-positive; median: 164, range: 119–227, in anti-Ro/SSA-negative; median: 190, range: 142–338, p = 0.03). In SLE patients, ApoCell-phagocytosis by monocytes (ApoCell-PhI values) correlated inversely with SLEDAI scores (r = −0.627, p = 0.0008, **Figure 1C**) and to be significantly impaired among patients with serum anti-Ro/SSA antibodies (in anti-Ro/SSA-positive; median: 149, range: 47–191, in anti-Ro/SSA-negative; median: 194, range: 136–308, p = 0.002), anti-dsDNA antibodies (in anti-dsDNA-positive; median: 154, range: 110–213, in anti-dsDNA-negative; median: 194, range: 47–308, p = 0.02) and low C3 complement levels (in those with low C3; median: 162, range: 47–212, in those with normal C3; median: 193, range: 123–308, p = 0.03).

### Impaired ApoCell-phagocytosis by MDM preparations of SS and SLE patients

The in-vitro differentiation of monocytes to macrophages (MDM) represents a surrogate for tissue macrophages [28] and a convenient means for the study of ApoCell-phagocytosis in the absence of direct influence of serum factors. In this context, ApoCell-phagocytosis was comparatively investigated in preparations of MDM derived from HBD and from SS and SLE patients. All HBD studied manifested substantial ApoCell-phagocytosis by MDM, whereas a significant number of SS and SLE cases showed impaired ApoCell-phagocytosis (ApoCell-PhI values less than two SD below the mean of HBD; in 4/9 of SS and in 7/10 of SLE) (**Figure 1D**). Parallel ApoCell-phagocytosis assays of peripheral blood monocytes from these individuals revealed significant agreement with those of MDM, being normal in all HBD (10/10), but impaired in the majority of patients (in 9/9 of SS and in 8/10 of SLE). In these patients, the occurrence of deficient ApoCell-phagocytosis by MDM always (in 11/11 of cases) correlated with decreased ApoCell-phagocytosis by peripheral blood monocytes. However, in 6 MDM preparations from patients (5 with SS and 1 with SLE), normal ApoCell-phagocytosis was associated with defective uptake by peripheral blood monocytes, a fact which likely suggests the influence of serum factors that operated only in the peripheral blood assays.

### ApoCell-phagocytosis by healthy monocytes is inefficiently supported by sera from SS and SLE patients

In preliminary experiments, the addition of serum specimens from healthy individuals was found to promote the ApoCell-phagocytosis by healthy peripheral blood monocytes. In fact, the replacement of healthy serum by DMEM resulted in significantly lower uptake of apoptotic cells (n = 13; median reduction: 48.0%, range: 34.2–56.9%, p = 0.001). In addition, the heat-treatment of HBD sera (n = 6) was also found to result in considerable reduction of ApoCell-phagocytosis by healthy monocytes (median reduction: 22.9%, range: 18.9–45.8%, p = 0.004), indicating the physiologic participation of heat-labile serum factors in the ingestion of apoptotic cells by monocytes.

In cross-admixture experiments, the application of apoptotic cells pre-treated with sera from SS and SLE patients (but not RA patients) resulted in significantly reduced ApoCell-phagocytosis by healthy peripheral blood monocytes (both for p<0.0001), compared to pretreatment with sera from HBD (**Figure 1E** **)**. In those experiments, ApoCell-PhI values obtained following the pretreatments with SS and SLE sera showed positive correlations with C3 and C4 complement levels (for SS sera; r = 0.510, p = 0.007 and r = 0.447, p = 0.02, respectively, for SLE sera; r = 0.467, p = 0.03 and r = 0.546, p = 0.008, respectively). Among SS and SLE sera used, those with reactivity to Ro/SSA and to native histones, respectively, showed significantly decreased ApoCell-phagocytosis (among SS sera: the anti-Ro/SSA-positive; median: 206, range: 118–320, the anti-Ro/SSA-negative; median: 356, range: 289–462, p = 0.0008, among SLE sera: the anti-histone-positive; median: 255, range: 209–300, the anti-histone-negative; median: 327, range: 245–450, p = 0.02).

Additional cross-admixture ApoCell-phagocytosis assays were also performed using peripheral blood monocytes obtained from selected SS and SLE patients with deficient ApoCell-phagocytosis (6 from SS and 3 from SLE patients) and apoptotic cells pre-treated with either the autologous serum or serum from a HBD. In these experiments, the pre-treatment of apoptotic cells with HBD serum was found to increase significantly ApoCell-phagocytosis values over those obtained with the autologous sera (median increase: 21.8%, range: 18.7–79.9%, p = 0.02). In fact, the incubation of apoptotic cells with HBD serum was found to confer normal ApoCell-phagocytosis values to the monocytes of 4/6 SS patients and of 1/3 SLE patients studied, a finding which suggests the key contribution of serum factor(s) in the aberrations observed, in a loss-of-function and/or a gain-of-function mode.

### Purified serum IgG from SS and SLE patients display inhibitory activity against ApoCell-phagocytosis that correlates with its binding to apoptotic cells

Sera from autoimmune patients (SS; n = 20, SLE; n = 14, RA; n = 5) and HBD (n = 12) were screened by flow cytometry for IgM and IgG immunoglobulin reactivity to early apoptotic cells. Such analyses had revealed that the levels of IgM anti-ApoCell antibodies were significantly decreased in the sera of SS patients (median IgM anti-ApoCell binding index [range]: 1.34 [0.22–5.05]) compared to HBD (3.26 [1.44–5.97], p = 0.0079), but not in those from SLE and RA patients (SLE: 4.91 [0.60–8.50], RA: 2.48 [1.58–3.28], differences not significant). In direct contrast, significantly increased levels of IgG anti-ApoCell antibodies were observed in the sera of SS and SLE patients (median IgG anti-ApoCell binding index [range]; SS: 1.70 [0.33–6.63], SLE: 1.57 [0.41–5.13]), compared to HBD (0.54 [0.38–0.73], for p = 0.035 and p = 0.001, respectively), but not in those from RA patients (0.70 [0.55–1.17], difference not significant). Rheumatoid factor positivity was not found to influence the IgG or IgM anti-ApoCell binding assay results (data not shown).

**Figure 2:**
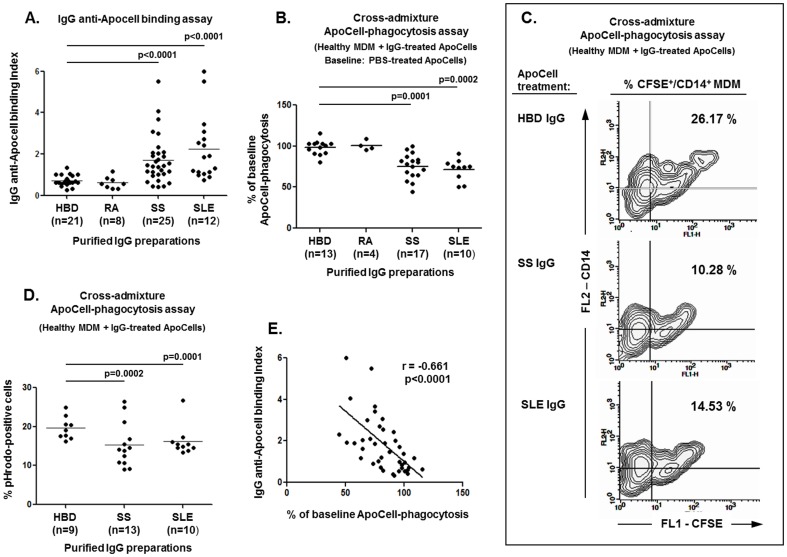
Inhibitory effect of IgG on ApoCell-phagocytosis. Purified serum IgG preparations from SS and SLE patients display inhibitory activity on ApoCell-phagocytosis by healthy MDM that correlates with its binding activity to early apoptotic cells. **A.** Purified serum IgG preparations from SS and SLE patients (but not RA) display significantly increased binding to early apoptotic cells. Binding index was normalized and expressed as fold increase over the binding of a purified IgG preparation from a HBD used in all experiments. **B–D**. Cross-admixture ApoCell-phagocytosis experiments demonstrating that the pretreatment of early apoptotic cells with purified serum IgG preparations derived from SS and SLE (but not from RA) results in decreased ApoCell-phagocytosis by healthy MDM, as compared to treatment with IgG from HBD. In **B**, data are expressed as percent of baseline ApoCell-phagocytosis values (e.g. treatment of apoptotic cells with PBS only, considered as 100%). Similar results were obtained by slightly different experimental setups assaying the ingestion of CFSE-labelled IgG-pretreated apoptotic cells by electronically gated CD14-stained MDM in dual-color flow cytometry (**C**; representative results from 3 independent experiments) or the uptake of pHrodo-SE-labelled apoptotic cells in single-color flow cytometry (**D**). **E**. Highly significant inverse correlation between the rates of ApoCell-phagocytosis obtained by the various purified serum IgG preparations used (shown in A) and the levels of anti-ApoCell antibodies in those preparations (shown in A).

The rates of ApoCell-phagocytosis that were observed in the cross-admixture experiments presented above (application of healthy peripheral blood monocytes and sera-pretreated apoptotic cells) correlated inversely and highly significantly with the levels of IgG anti-ApoCell antibodies in those sera, (r = −0.631, p = 0.0009), but not those of IgM (r = 0.284, difference not significant). Therefore, to address whether the IgG anti-ApoCell antibodies that were present in the sera of SS and SLE patients may interfere adversely with the ingestion of apoptotic cells, serum IgG immunoglobulins were purified from the sera of autoimmune patients and controls and subsequently analyzed for anti-ApoCell reactivity, as well as in cross-admixture ApoCell-phagocytosis assays. The levels of anti-ApoCell antibodies were significantly increased in the IgG preparations derived from SS and SLE patients (both for p<0.0001, compared to HBD), but in not those from RA (**Figure 2A**). In addition, anti-ApoCell levels were significantly higher among anti-Ro/SSA antibody-positive patients (median: 1.833, range: 0.426-5.500) compared to antibody-negative ones, (median: 1.030, range: 0.445–2.020, p = 0.019). In cross-admixture ApoCell-phagocytosis assays, the application of healthy MDM and purified IgG-pretreated early apoptotic cells had revealed significantly reduced ApoCell-phagocytosis upon the application of IgG preparations derived from SS and SLE patients (for p<0.0001 and p = 0.0002, respectively, compared to HBD) but not from RA (**Figure 2B & 2C**) suggesting the inhibitory effect of IgG anti-ApoCell antibodies in SS and SLE on ApoCell-phagocytosis. The significantly impaired engulfment of apoptotic cells following their pretreatment with purified IgG from SS and SLE patients compared to that from HBD, was also demonstrated by comparative ApoCell-phagocytosis assays using pHrodo-SE-labelled apoptotic cells (**Figure 2D**). Importantly, highly significant inverse correlation was found between the rate of ApoCell-phagocytosis obtained by the various IgG preparations used and the levels of anti-ApoCell antibodies in those preparations (r = −0.661, p<0.0001, **Figure 2E**). Similar inverse correlation was observed between the ApoCell-PhI values obtained with the application of whole sera in the cross-admixture experiments described earlier, and the anti-ApoCell levels that were detected in purified IgG preparations from the respective sera used (in 25 sera; 8 from SS, 7 from SLE, 4 from RA patients and 6 from HBD, r = −0.595, p = 0.0017).

### Significantly impaired MB-phagocytosis by peripheral blood monocytes of SS and SLE patients

**Figure 3:**
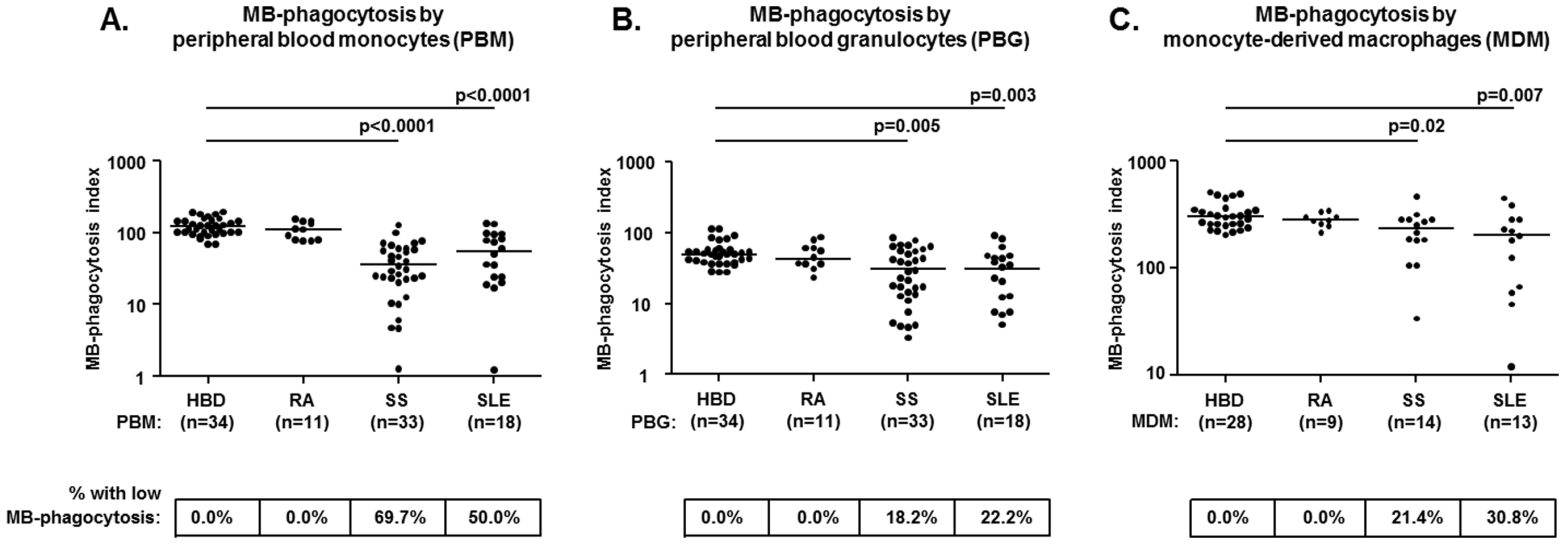
Impaired MB-phagocytosis by peripheral blood (PB) phagocytes (A; monocytes B; granulocytes) and by monocyte-derived macrophages (MDM, C) obtained from SS and SLE patients, but not from RA patients. The horizontal lines indicate the median levels in each group, whereas the numbers in boxes indicate the percentages of individuals with decreased MB-phagocytosis, as defined by the presence of MB-PhI values that were two standard deviations below the corresponding mean of HBD. Statistically significant comparisons of patient groups to HBD are shown.

RA patients displayed similar MB-phagocytosis indices to those of HBD. In direct contrast, both SS and SLE groups were found to manifest highly significantly decreased levels of MB-phagocytosis by granulocytes and by monocytes compared to HBD, as well as to RA (**Figure 3A & 3B**). Conversely, there were no differences of MB-phagocytosis indices between SS and SLE patients for either granulocytes or monocytes (**Figure 3A & 3B**). The overall analysis of phagocytosis index values had revealed a highly significant correlation between the levels of MB-phagocytosis and ApoCell-phagocytosis by monocytes in the various groups studied (r = 0.432, p = 0.001).

Among the various clinical and serological parameters examined, the degree of MB-phagocytosis by monocytes was found to positively correlate with the C4 serum complement levels in both SS and SLE patients (SS; r = 0.377, p = 0.03, SLE; r = 0.520, p = 0.03). Furthermore, in SS patients, the occurrence of extraglandular disease correlated with significantly lower levels of MB-phagocytosis by granulocytes (glandular SS; median = 41, range: 5–84, extraglandular SS; median = 14, range: 3–63, p = 0.003), as well as by monocytes (glandular SS; median = 58, range: 11–132, extraglandular SS; median = 24, range: 1–87, p = 0.002). Finally, MB-phagocytosis indices in SS patients also inversely correlated with ESSDAI (for granulocytes; r = −0.432, p = 0.01, for monocytes; r = −0.520, p = 0.002) and SSDDI (for granulocytes; r = −0.355, p = 0.02, for monocytes; r = −0.475, p = 0.006).

### Impaired MB-phagocytosis by MDM preparations of SS and SLE patients

The study of MB-phagocytosis by MDM had yielded similar results between RA patients and HBD, whereas significantly decreased MB-phagocytosis was observed in both SS and SLE patient groups, compared to HBD (p = 0.01 and p = 0.004, respectively), as well as to RA patients (**Figure 3C**). The overall analysis of MB-PhI values had revealed a significant correlation of the levels of MB-phagocytosis by MDM with those obtained by peripheral blood monocytes in the various groups studied (r = 0.552, p = 0.01). In SS patients, MB-phagocytosis by MDM was marginally lower among patients with extraglandular disease (median = 184, range: 34–312), compared to those with disease confined in glands (median = 282, range: 252–468, p = 0.07), as well as among patients with type-I SS (median = 183, range: 34–312), compared to those without (median = 281, range: 252–468, p = 0.06). Among the SLE patients studied, the occurrence of decreased MB-phagocytosis by MDM was not found to correlate significantly with any of the clinical and serological parameters examined.

## Discussion

Several previous studies had documented that the monocytes of SLE patients manifest reduced clearance of various targets [29], including defective efferocytosis [9], [11], [17], [26]. In fact, the accumulation of apoptotic cells in lymph node and skin biopsies of SLE patients is thought to signify the deficient phagocytic clearance of apoptotic cells in vivo [30]. These data had provided a mechanistic explanation for the development of chronic inflammatory and autoimmune reactions in SLE patients, via the progression of uncleared apoptotic cells to the state of secondary necrosis and the release thereof of alarmins and modified self antigens that activate innate and acquired immune system [31]. In fact, experimental mice that are deficient for molecules involved in the phagocytosis of apoptotic cells display defective efferocytosis, as well as features of SLE, such as the development of antinuclear antibodies and glomerulonephritis [32]. In the same context, lupus-prone strains of mice are reported to display decreased phagocytosis of apoptotic cells by macrophages [33].

SLE and SS are immunologically similar disorders in several respects [18], therefore in this study we sought to evaluate directly the capacity of the peripheral blood monocytes of SS and SLE patients for uptake of early apoptotic cells, employing simple and reproducible ex-vivo ApoCell-phagocytosis assays. In addition, several lines of experimental evidence from mice and human studies indicate that apoptosis plays a crucial role in the pathophysiology of SS [34]–[36], whereas SS-related autoantigens, such as Ro(SSA) and La(SSB), have been shown to be clustered at the surface of apoptotic cells [37].

In good concordance with previous studies [9], [26], our findings indicate that compared to healthy individuals, approximately half of the SLE patients tested manifested significantly impaired ApoCell-phagocytosis by monocytes. In addition, this study provides first evidence that, in a manner similar to SLE, deficient uptake of early apoptotic cells by monocytes also characterizes a significant proportion of SS patients, whereas such aberration is not apparently present among RA patients. Interestingly, previous studies of experimental animal models had indicated decreased ApoCell-phagocytosis by macrophages not only in lupus-prone strains of mice [33] but also in mice susceptible to SS-like sialadenitis [38]. In addition, defective efferocytosis has been described to occur in the heart of fetuses of certain SS and SLE patients owing to aberrant opsonization of apoptotic cells by maternal IgG anti-Ro/SSA and anti-La/SSB antibodies [39]. Furthermore, in the present study the rates of ApoCell-phagocytosis in SS and SLE patients correlated inversely and highly significantly with the activity indices of these disorders. Although larger cohort studies with a wide sampling of patients are needed, our findings support aberrant efferocytosis as an important pathogenetic mechanism for both SS and SLE and as a promising field of search for novel biomarkers for these diseases. In fact, the inverse correlation between deficient ApoCell-phagocytosis and disease activity has been also previously observed in SLE patients [11].

The underlying cause of defective efferocytosis in SLE has been attributed to the presence of intrinsic functional defects in patients' phagocytes [9], [11], [17], [26] and/or aberrant serum factors [16], [17]. Our findings mainly indicate that serum factors in SS and SLE patients are mostly responsible for the observed impairment of ApoCell-phagocytosis; however, certain lines of evidence may be also supportive to the notion of disordered function of patients' phagocytes per se. First, in line with previous reports in SLE patients [9], [26], our data indicate that ApoCell-phagocytosis is deficient in several MDM preparations that were derived from SS and SLE patients by cultivation in the absence of patients' sera, a fact that may at least partly support a model of intrinsically defective monocyte in these disorders. Alternatively, the impaired capacity of phagocytes of SS and SLE patients for uptake of prey may be viewed as a result of cellular activation taking place in vivo, a notion probably also implied by the significant correlation between the findings in the various phagocytosis assays and the activity indices of these diseases. Finally, the notably low capacity of SS and SLE patients for phagocytosis of particulate targets by blood-borne monocytes and MDM that was observed may reflect an overall dysfunction of phagocytes in the proper engulfment of certain preys in these disorders. Interestingly, deficient phagocytosis of particulate targets is also reported in primary biliary cirrhosis [40], a disease with striking clinicopathologic similarities to SS [41]. On the other hand, it should be noticed that in sharp contrast to the above deficient phagocytic capacities, the blood-borne monocytes of SS and SLE were found remarkably hyperfunctional in the phagocytosis of necrotic cell debris [42].

The addition of healthy serum was found to facilitate significantly the ingestion of apoptotic cells by blood-borne monocytes derived from healthy individuals, as well as from SLE or SS patients. These findings are in good agreement with the previously observed capacity of sera from healthy individuals to restore the phagocytic ability of macrophages from SLE patients [11]. In addition, ApoCell-phagocytosis by healthy monocytes was presently shown to be severely impaired following the substitution of HBD sera by sera derived from SS and SLE patients. Consistent with previous observations [17], the sera from approximately 80% of SLE patients studied were not as efficient as healthy sera in supporting of ApoCell-phagocytosis by normal blood-borne monocytes. In this study, we present first evidence that such incapacity is also manifested by the vast majority of sera from SS patients studied. The precise nature of this aberration in SS and SLE patients is unclear. Normally, several serum proteins (such as complement C1q, IgM, C-reactive protein, serum amyloid protein, milk fat globule-EGF factor 8 protein [MFG-e8] and mannose-binding lectin) attach to apoptotic cells and induce the deposition of C3 and its degradation products C3b and iC3b, thus enhancing efferocytosis by phagocytes via recognition by complement receptors CR3 and CR4 [43]. In this context, mice lacking such bridging molecules, such as MFG-e8, Mer or C1q are reported to develop lupus-like manifestations associated with inefficient removal of apoptotic cells [32]. In line to these observations, several quantitative and functional aberrations of the above opsonins have been described in SLE and SS patients, including hypocomplementemia, which is considered as one of the major immunological markers and of key clinical importance for both disorders [44], [45]. In fact, an intact classical complement pathway is essential for the phagocytic removal of apoptotic cells [46], whereas the deficient ApoCell-phagocytosis in SLE patients has been previously attributed to defective opsonization of apoptotic cells by aberrantly low levels of C1q, C3, and C4 complement proteins [17]. Our results from the cross-admixture experiments also implicate the role of aberrant serum complement function in SS and SLE patients in the observed defect of ApoCell-phagocytosis. The capacity of the SS and SLE patients' sera to assist ApoCell-phagocytosis by healthy monocytes was found to inversely correlate with C3 and C4 complement levels in these sera. In line with these observations, the decomplementation of normal sera by heat-inactivation largely diminished their capacity to support ApoCell-phagocytosis by healthy monocytes.

Most remarkably however, our results had indicated that the inhibitory component of SS and SLE sera to ApoCell-phagocytosis largely resides in the IgG immunoglobulin fraction. Serum IgG preparations from SS and SLE patients (but not from controls) were very frequently found to possess significant inhibitory activity on ApoCell-phagocytosis by normal monocytes, as well as enhanced binding capacity for the surface of early apoptotic cells. Moreover, the rate of ApoCell-phagocytosis obtained by the various IgG preparations used in cross-admixture experiments was presently found to correlate significantly in an inverse manner with the levels of anti-ApoCell antibodies in those preparations. In fact, serum antibodies against late, but not against early apoptotic cells, have been previously reported in the serum of SLE patients and to prevent the uptake of opsonized late apoptotic cells by phagocytes [16].

The nature of IgG antibodies to early apoptotic cells that we have detected in the sera of SS and SLE patients is presently unclear. In the recent years, growing evidence indicates that various types of serum autoantibodies may interfere with the clearance of apoptotic cells by phagocytes. Such autoantibody responses may involve reactivities against surface membranous molecules of the apoptotic cells and/or the phagocytes [16], [47], as well as against bridging molecules that normally facilitate ApoCell-phagocytosis [48]. Recently, the occurrence of anti-C3 complement autoantibodies in SLE patients has been shown to react to C3 bound on apoptotic cells and thus to interfere with the proper C3-mediated recognition and clearance of apoptotic cells by phagocytes [49]. Anti-nuclear antibodies, which are a hallmark feature of SLE and SS, may also hamper the uptake of apoptotic cells by phagocytes. In fact, autoantibodies from SLE and SS patients are reported to opsonize late apoptotic cells and to inhibit their uptake by macrophages via an Fc-gamma receptor-dependent mechanism [16]. In line with these observations, our results from cross-admixture experiments had also indicated that the occurrence of specific anti-nuclear antibodies in the sera of SLE (anti-histones) and SS patients (anti-Ro/SSA) is associated with failure to support the ApoCell-phagocytosis by healthy monocytes, whereas we have found increased IgG binding activity to early apoptotic cells among anti-Ro/SSA-positive sera. Notably, apoptotic cells are reportedly characterized by surface translocation of various nuclear constituents, including the ribonucleoproteins Ro/SSA and La/SSB [37], which represent the major targets of autoimmune responses in SS. Importantly also, IgG anti-Ro/SSA and anti-La/SSB antibodies have been previously shown to opsonize in-vitro apoptotic cardiocytes and thus to inhibit their clearance by phagocytes [39]. To this end, detailed autoantibody depletion and antigen inhibition experiments are in progress in this laboratory to delineate the characteristics and role of particular antibody specificities in the clearance of apoptotic cells. On the other hand, loss-of-function processes involving the natural IgM immunoglobulins of SS patients may also have a role, as indicated by the occurrence of significantly decreased IgM anti-ApoCell levels that we observed in the SS patients, compared to healthy individuals. In fact, the important role of natural IgM antibodies for the clearance of apoptotic cells, microbes and various small particles is well-described [50].

In conclusion, this study demonstrates that in a manner similar and comparable to SLE, a significant portion of SS patients is characterized by impaired uptake of early apoptotic cells, as well as of particulate targets by blood-borne phagocytes and macrophages that apparently involves both loss-of-function and gain-of-function processes. Importantly, these aberrations were found to correlate with various clinico-serologic disease indices of SS and SLE, and thus may represent promising areas of search for novel biomarkers for these disorders. The defective clearance of apoptotic cells in SS and SLE appears primarily to depend on serologic aberrations, such as the occurrence of inhibitory IgG anti-ApoCell antibodies and hypocomplementemia, and secondarily on the dysfunction of phagocytes. Such failure of efferocytosis may lead to the accumulation of immunogenic and inflammagenic secondary necrotic cells and debris and the perpetuation thereof of a vicious cycle of inflammatory and autoimmune reactions. In fact, we have recently demonstrated that, SS and SLE patients also manifest impaired serum-mediated degradation of necrotic cell debris that leads to increased amounts of circulating nuclear material and their massive uptake by blood-borne phagocytes [42]. Altogether, these aberrations may represent major causes of the inflammatory and autoimmune reactions that characterize SS and SLE, and may thus hold key roles in the pathogenesis of these disorders.
